# Chemical or genetic Pin1 inhibition exerts potent anticancer activity against hepatocellular carcinoma by blocking multiple cancer-driving pathways

**DOI:** 10.1038/srep43639

**Published:** 2017-03-06

**Authors:** Xin-Hua Liao, Arina Li Zhang, Min Zheng, Mei-Qing Li, Champ Peng Chen, Huijuan Xu, Qing-Song Chu, Dayun Yang, Wenxian Lu, Ting-Fen Tsai, Hekun Liu, Xiao Zhen Zhou, Kun Ping Lu

**Affiliations:** 1 Fujian Key Laboratory for Translational Research in Cancer and Neurodegenerative Diseases, Institute for Translational Medicine, Fujian Medical University, Fuzhou, Fujian 350122, China; 2 Key Laboratory of Ministry of Education for Gastrointestinal Cancer, Fujian Medical University, Fuzhou, Fujian 350122, China; 3 Program in Molecular Medicine, National Yang-Ming University, Taipei 112, Taiwan; 4 Division of Translational Therapeutics, Department of Medicine and Cancer Research Institute, Beth Israel Deaconess Medical Center, Harvard Medical School, Boston, MA 02215, USA

## Abstract

Hepatocellular carcinoma (HCC) is one of the most prevalent and malignant cancers with high inter- and intra-tumor heterogeneity. A central common signaling mechanism in cancer is proline-directed phosphorylation, which is further regulated by the unique proline isomerase Pin1. Pin1 is prevalently overexpressed in human cancers including ~70% of HCC, and promotes tumorigenesis by activating multiple cancer-driving pathways. However, it was challenging to evaluate the significance of targeting Pin1 in cancer treatment until the recent identification of all-trans retinoic acid (ATRA) as a Pin1 inhibitor. Here we systematically investigate functions of Pin1 and its inhibitor ATRA in the development and treatment of HCC. Pin1 knockdown potently inhibited HCC cell proliferation and tumor growth in mice. ATRA-induced Pin1 degradation inhibited the growth of HCC cells, although at a higher IC50 as compared with breast cancer cells, likely due to more active ATRA metabolism in liver cells. Indeed, inhibition of ATRA metabolism enhanced the sensitivity of HCC cells to ATRA. Moreover, slow-releasing ATRA potently and dose-dependently inhibited HCC growth in mice. Finally, chemical or genetic Pin1 ablation blocked multiple cancer-driving pathways simultaneously in HCC cells. Thus, targeting Pin1 offers a promising therapeutic approach to simultaneously stop multiple cancer-driving pathways in HCC.

Hepatocellular carcinoma (HCC) is highly prevalent especially in Asia and sub-Saharan Africa, and has an increasing trend in previously low incidence areas such as the US and Europe^1^. Despite significant advance in understanding molecular mechanisms of HCC tumorigenesis, there is still lack of efficacious therapeutics. Cytotoxic chemotherapeutic agents show little improvement of overall survival^2^, while the only FDA-approved HCC targeted drug Sorafenib increases overall survival modestly by 2.8 months in advanced cancer patients^3^. As a result, the majority of HCC patients die within 9–12 months after diagnosis and HCC has moved up the rank from the third most common cause of cancer death to the second according to the recently released *World Cancer Report 2014*, even though it is the sixth most common cancer^4^.

Targeted drugs have changed cancer treatment, but are often ineffective against aggressive solid tumors due to the activation and feedback of multiple interactive and/or redundant cancer-driving pathways^5^,^6^,^7^,^8^,^9^. A central common signaling mechanism in cell proliferation and transformation is proline-directed phosphorylation that is regulated by many kinases and phosphatases, but further controlled by a unique proline isomerase Pin1^10^,^11^,^12^. Pin1 is prevalently overexpressed or overactivated in many cancer types^13^, Pin1 promotes cancer and cancer stem cells tumorigenesis by turning on more than 40 oncogenes/growth-promoting factors and off more than 20 tumor suppresses/growth-inhibitory factors^12^. Interestingly, although Pin1 is important for cancer cells growth, it is dispensable for normal cell growth. Pin1^−/−^ mice develop normally and do not exhibit obvious defects at young ages^14^. However, these Pin1 null mice are highly resistant to Ras, Neu/HER2 or mutant p53-induced breast cancer^15^,^16^, Myc-induced Burkitt’s lymphoma^17^ or Notch3-induced T-cell acute lymphoblastic leukemia^18^, demonstrating an essential role for Pin1 in tumorigenesis of many cancer types. Thus, targeting Pin1 represents a novel anticancer strategy to block multiple cancer pathways simultaneously without general toxic effects on normal tissues. However, because the available Pin1 inhibitors lacked the required specificity and/or potency, or cannot enter cells^19^,^20^,^21^, it was challenging to evaluate the significance of targeting Pin1 for cancer treatment until our discovery of all-trans retinoic acid (ATRA) as a Pin1 inhibitor^22^.

Use of ATRA for acute promyelocytic leukemia (APL) is considered the first example of modern targeted cancer therapy^23^,^24^,^25^,^26^,^27^, but how it causes driven fusion oncogene PML–RAR-α degradation and inhibition of self-renewal of leukemia stem cells had remained elusive^28^,^29^. Unexpectedly, ATRA was discovered as a Pin1 inhibitor from a high throughput screening. ATRA was proved to be a potent submicromolar Pin1 inhibitor that specifically binds to, inhibits and ultimately degrades the active Pin1 selectively without cross-reacting with other isomerase members^22^. ATRA also shows detectable but not striking anticancer activity against many solid tumors^30^,^31^,^32^, which is likely due to its light sensitivity and a short half-life of 45 minutes in humans^33^. However, using slow-releasing ATRA pellets to maintain constant blood drug levels, we have shown that ATRA exerts potent anticancer activity against both APL and aggressive triple negative breast cancer by inhibiting and ablating Pin1 and thereby turning off and on numerous oncogenes and tumor suppressors, respectively, at the same time^22^. These results indicate that Pin1 inhibitors have the unique and promising property to effectively block multiple cancer-driving pathways at once^12^,^22^.

The above property of Pin1 inhibitors are desirable for treating aggressive solid tumors such as HCC, because HCC caused by various etiologies has a high intertumor and intratumor heterogeneity, with multiple interactive and/or redundant cancer-driving pathways being activated simultaneously in the same patient^34^,^35^,^36^,^37^. Furthermore, Pin1 is overexpressed in about 70% of human HCC patients and it promotes heptocarcinogenesis in cell line PLC/PRF/5^38^,^39^,^40^,^41^. Pin1 might play different roles in HCC cells depending on status of TP53 gene mutation^42^. Pin1 single nucleotide promoter and exon polymorphisms are associated with the susceptibility of HBV-related HCC^43^. Interestingly, Pin1 also physically interacts with and increases hepatitis B virus X-protein (HBx) protein stability to enhance hepatocarcinogenesis^44^. These results suggest that Pin1 plays an important role in hepatocarcinogenesis, functioning as novel anticancer target. However, the significance of targeting Pin1 for HCC treatment is not clear.

Here, we show that either chemical or genetic Pin1 inhibition potently suppresses HCC growth by blocking multiple cancer-driving pathways. Silencing Pin1 expression in multiple human HCC cell lines by a validated shRNA not only potently inhibited HCC cell proliferation and migration, but also suppressed HCC tumor growth in mice. ATRA induced Pin1 protein degradation in HCC cells, although at a higher IC50 as compared with breast cancer cells, possibly due to a high ATRA metabolic enzyme in liver cells. Indeed, inhibition of such enzyme significantly enhanced the sensitivity of HCC cells to ATRA. Moreover, chemical or genetic ablation of Pin1 blocked multiple cancer-driving pathways simultaneously. Finally, slow-releasing ATRA potently inhibited HCC growth *in vivo* without obvious side effects. These results demonstrate that Pin1 is a promising therapeutic target in HCC and provide a further rationale for developing longer half-life ATRA or more potent and specific Pin1-targeted ATRA variants for treating HCC and other cancers.

## Results

### Pin1 knockdown suppresses cell proliferation in various human HCC cell lines

**Figure 1:**
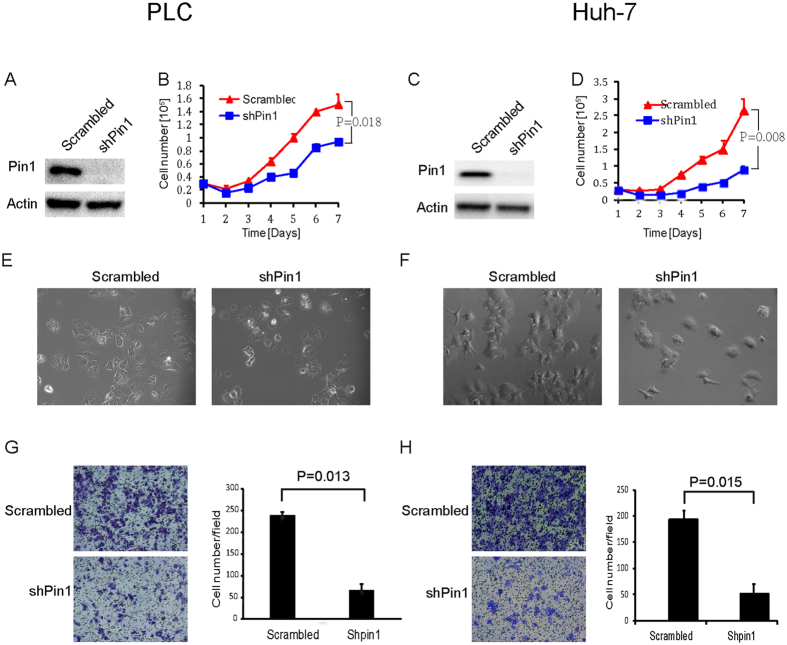
Pin1 knockdown suppresses the proliferation of human HCC cells *in vitro*. PLC/PRF/5 (**A**) or Huh-7 (**C**) cell lines were infected with lentiviruses expressing scrambled or Pin1 shRNA. Cell lysates were subject to western blot analysis with antibodies against Pin1 or internal control Actin. Growth curves of PLC/PRF/5 (**B**) and Huh-7 (**D**) cell lines with Pin1 knockdown were plotted over time, based on the cell numbers counted daily. 3 × 10^4^ cells for both cell lines were placed in each well of 12-well plate with triplicate for each time point. Error bars represent standard deviations (N = 3). *p* values were derived from the cell numbers at the end point. (**E**,**F**) Morphological change of PLC/PRF/5 (**E**) and Huh-7 (**F**) cells after Pin1 knockdown. (**G**,**H**) Pin1 knockdown affects cells migration in PLC/PRF/5 (G) and Huh-7 (**H**), as evaluated by transwell assay. 5 × 10^4^ cells for both cell lines were used for this assay. Error bars represent standard deviations (n = 2).

Pin1 has been shown to be overexpressed in about 70% human HCC^38^, but its therapeutic potential in treating HCC is still not clear. To determine the importance of Pin1 in HCC, we first examined the effects of Pin1 loss of function by genetically suppressing Pin1 expression in various human HCC cell lines using lentiviruses expressing a validated Pin1 shRNA^45^. PLC/PRF/5 (HBV-positive) (Fig. 1A,B,E), Huh-7 (HBV-negative) (Fig. 1C,D,F) and several other HCC cells (including Hep3B and Sk-Hep-1, data not shown) became unhealthy after infection with lentiviruses expressing Pin1 shRNA, as compared with scrambled shRNA. PLC/PRF/5 cells growth was significantly retarded, as illustrated by growth curves over time (Fig. 1B). Huh-7 cells showed similar dependency on Pin1 but to a less extent (Fig. 1D). After Pin1 knockdown, overall cell morphology of both PLC/PRF/5 and Huh-7 cells were dramatically changed, with loss of protruding pseudopodia (Fig. 1E,F). Consistent with these morphological changes are that migration of Pin1 knockdown HCC cells was significantly suppressed, as shown in transwell assay (Fig. 1G,H). Since lack of Pin1 has little effect on normal tissues and cells, as demonstrated in Pin1^−/−^ mice and MEFs^14^, these results indicate that human HCC cells likely develop addiction to Pin1, demonstrating a pivotal role for Pin1 in HCC cells *in vitro*.

### Pin1 knockdown suppresses HCC growth *in vivo*

**Figure 2:**
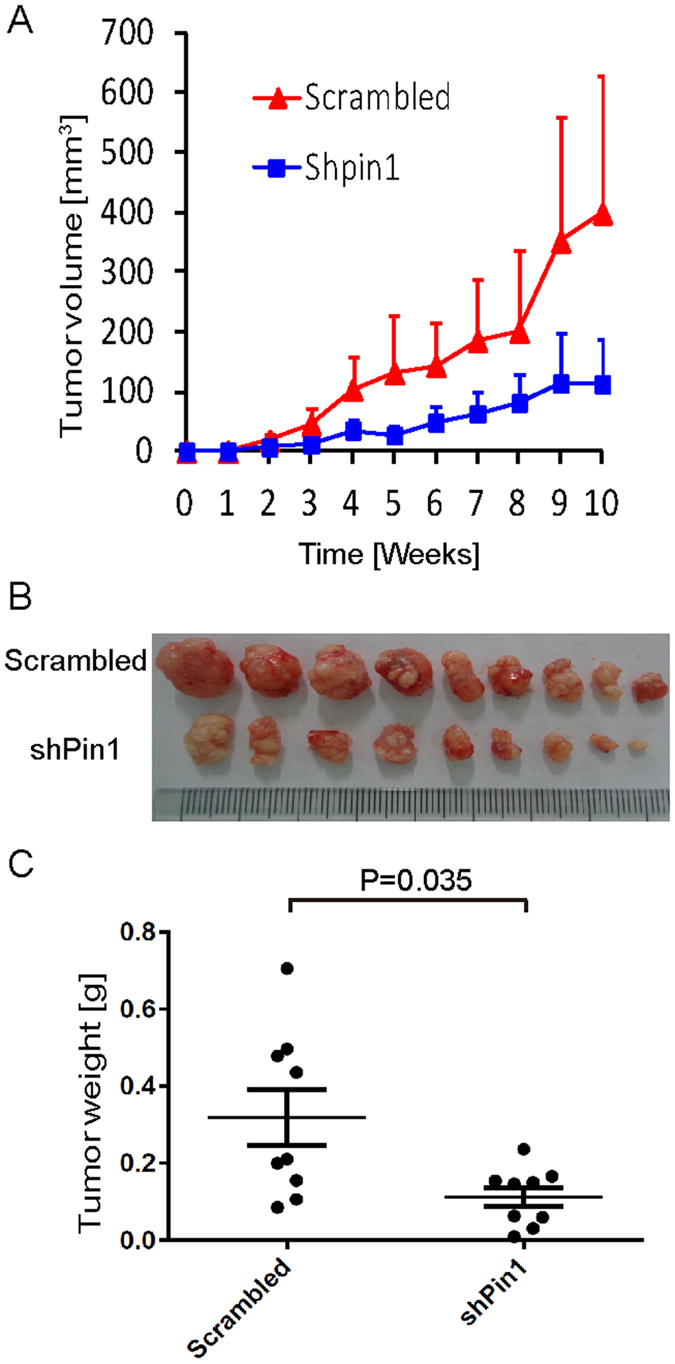
Pin1 knockdown suppresses tumor growth of human HCC cells *in vivo*. 2 × 10^5^ Huh-7 cells infected with lentiviruses expressing scrambled or Pin1 shRNA were inoculated subcutaneously into nude mice. (**A**) Huh-7 tumor volumes were measured weekly and the tumor growth curves were plotted over time. Error bars represent standard deviations. (**B**) Photographic illustration of 9 pairs of tumors harvested from nude mice at the end point (10 weeks). Each scale of the ruler represents 1 mm. (**C**) Weights of tumors harvested from nude mice at the end point. Error bar represents SEM (n = 9).

To examine whether Pin1 affects HCC tumorigenesis *in vivo*, we performed xenograft tumor growth assay in nude mice. After expansion *in vitro* (<6 passages after lentiviral infection), 2 × 10^6^ Huh-7 cells expressing Pin1 shRNA or scrambled shRNA were subcutaneously injected into the left and right flanks of the same mice, respectively. Tumor growth curves were established over time by measuring the tumor size every week. When tumor diameter reached 1.5 cm, all of tumors were dissected from the nude mice, photographed and weighed. The tumor growth was significantly suppressed after Pin1 knockdown, as illustrated by the tumor growth curve (Fig. 2A) and tumor weight (Fig. 2B,C) n = 9, P = 0.035). Thus, Pin1 represents a promising therapeutic target in HCC.

### ATRA promotes Pin1 degradation in HCC cell lines

**Figure 3:**
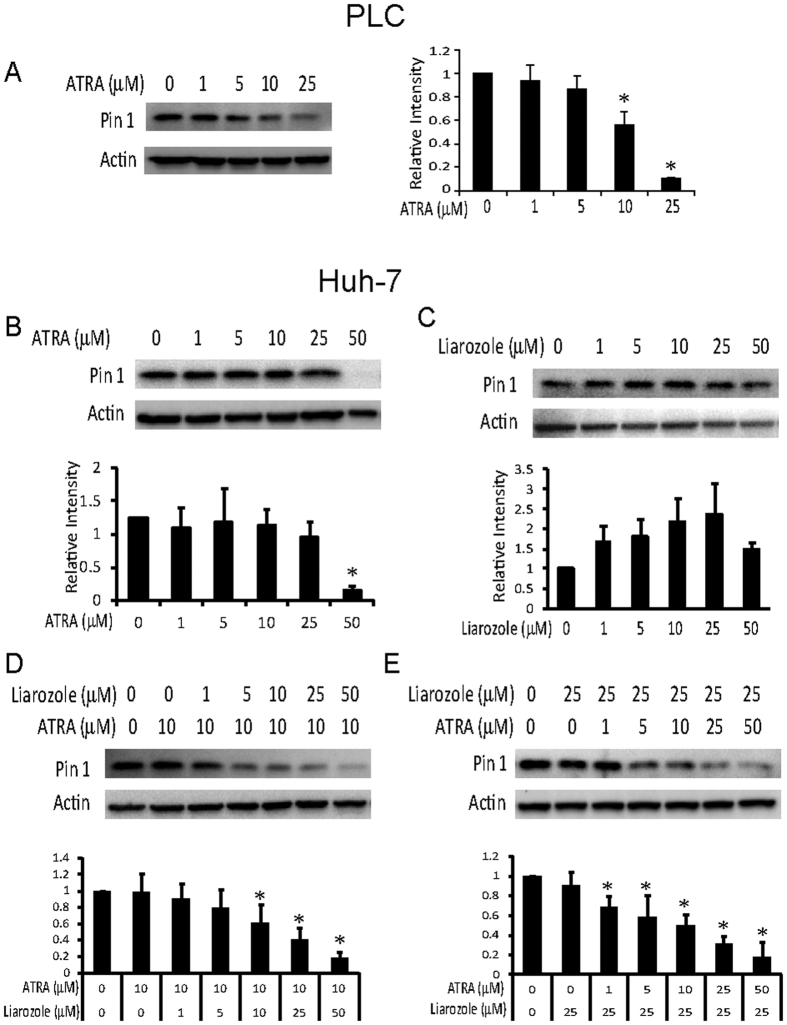
The Pin1 inhibitor ATRA induces Pin1 degradation in HCC cell lines. PLC/PRF/5 or Huh-7 cells were treated with chemicals for 72 hours, followed by subjecting cell lysates to immunoblotting with Pin1 antibody. (**A**) PLC/PRF/5 cells were treated with different doses of ATRA. (**B**,**C**) Huh-7 cells were treated with different doses of ATRA (**B**) or liarozole (**C**) alone. (**D**) Huh-7 cells were treated with fixed 10 μM ATRA combined with different doses of liarozole. (**E**) Huh-7 cells were treated with fixed 25 μM liarozole combined with different doses of ATRA. The graphics were derived from quantification of Pin1 relative intensity normalized with Actin intensity. The error bars represent standard deviations from two to four independent blots. *p < 0.05 vs control.

Since shRNA could have off-target effects and that it is still very challenging to deliver shRNA to tumors for cancer therapy, we used a small molecular Pin1 inhibitor, ATRA, which has been identified through a mechanism-based screening from a compound library and found to bind to the Pin1 active site and thereby induce degradation of active Pin1^22^. In PLC/PRF/5 HCC cells, ATRA began to induce Pin1 degradation at 5 μM (Fig. 3A), a little higher in comparison with ~1 μM in the breast cancer and APL cells^22^. However, in Huh-7 HCC cells, Pin1 was only degraded efficiently at 50 μM, a much higher dose in comparison with the breast cancer and APL cells (Fig. 3B). Since ATRA is known to be metabolized in the liver by enzymes including cytochrome P450^46^, lower sensitivity of Huh-7 HCC cells to ATRA might be due to high activity of ATRA metabolism. To examine this possibility, we used liarozole that inhibits the cytochrome P450-dependent 4-hydroxylation of retinoic acid, thereby reducing ATRA drug metabolism^46^. After Huh-7 HCC cells were treated with ATRA or liarozole or their combination for various times, Pin1 degradation was assayed. Although liarozole alone did not significantly induced Pin1 degradation and might even slightly elevate Pin1 protein levels (Fig. 3C), its combination with ATRA potently increased the ability of ATRA to induce Pin1 degradation (Fig. 3D,E). When the concentration of ATRA was fixed at 10 μM, Pin1 was degraded in a dose dependent manner depending on increasing concentrations of liarozole (Fig. 3D), with IC50 ~24.5 μM. In the other hand, when the concentration of liarozole was fixed at 25 μM, Pin1 was degraded in a dose dependent manner depending on increasing concentrations of ATRA (Fig. 3E), with IC50 ~7.3 μM. These results indicate that liarozole functions on Pin1 through stabilizing ATRA and further support the specificity of Pin1 degradation induced by ATRA. Taking together, these results demonstrate that ATRA targets Pin1 in HCC, as in APL and breast cancer^22^.

### ATRA inhibits HCC cells growth *in vitro*

**Figure 4:**
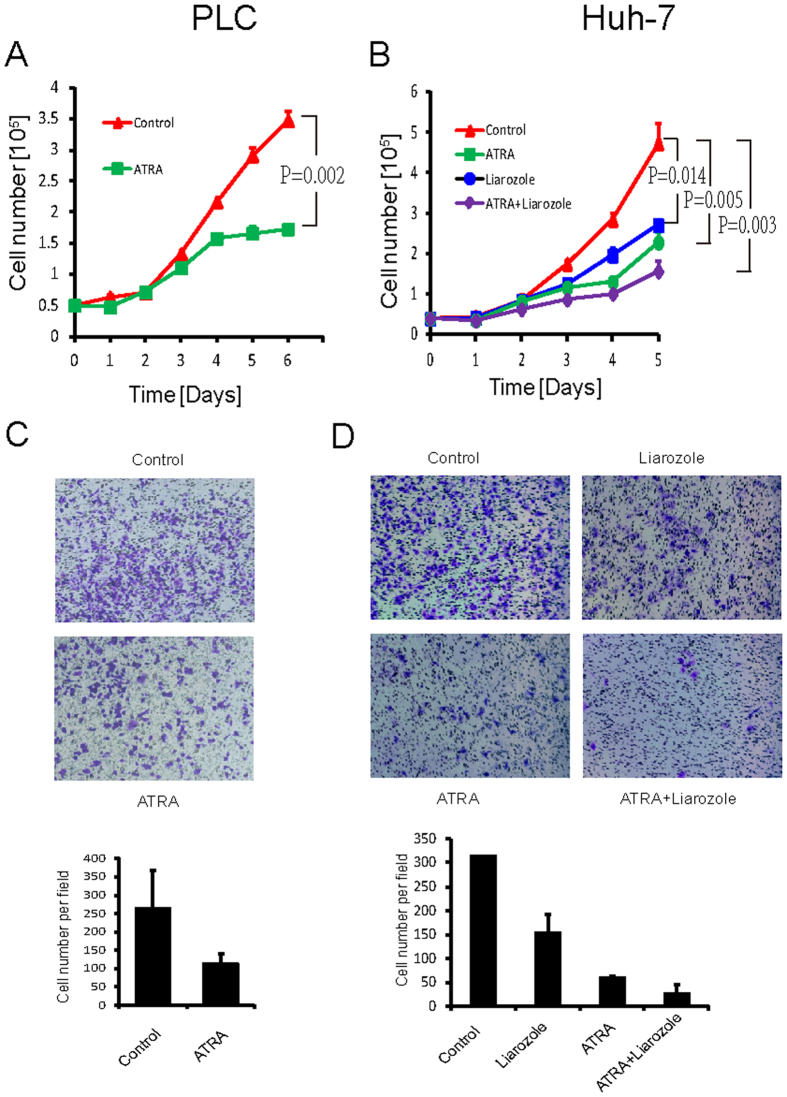
ATRA suppresses HCC cell growth and migration *in vitro*. (**A**,**B**) PLC/PRF/5 (**A**) or Huh-7 (**B**) cells were treated with 10 μM ATRA and/or 25 μM P450 inhibitor liarozole and the growth curves were plotted over time. 5 × 10^4^ of PLC/PRF/5 cells or 3 × 10^4^ of Huh-7 were placed in each well of 12-well plate with triplicate for each time point. Error bars represent standard deviations (n = 3). *p* values were derived from the cell numbers at the end point. (**C**,**D**) PLC/PRF/5 (**C**) or Huh-7 (**D**) cells were treated with ATRA and/or P450 inhibitor liarozole, as evaluated by transwell assay. 5 × 10^4^ cells for both cell lines were used for this assay. Error bars represent standard deviations (n = 2).

The above results indicate that Pin1 genetic knockdown inhibits HCC cells growth and that ATRA inhibits and degrades Pin1 in HCC cells. We then asked whether ATRA would mimic Pin1 genetic inhibition to suppress HCC cell growth. ATRA alone effectively suppressed the cell growth in PLC/PRF/5 cells (P = 0.002), and also showed inhibitory activity in Huh-7 cells (P = 0.005) (Fig. 4A). We then used liarozole to inhibit ATRA metabolism in Huh-7 cells, as described above. Although liarozole alone had some inhibitory effects (P = 0.014), its combination with ATRA further increased the ability of ATRA to inhibit cell proliferation of Huh-7 cells (P = 0.003) (Fig. 4B). Similarly, we examined ATRA effects on HCC cells migration. ATRA alone suppressed cells transwell both in PLC/PRF/5 and Huh-7 cell lines (Fig. 4C,D). A combination of ATRA with liarozole further increased the inhibitory effects on Huh-7 cells migration, which is consistent with the results that liarozole helps ATRA to induce Pin1 degradation in Huh-7 cells (Fig. 4D). These results show that the Pin1 inhibitor ATRA inhibits HCC cell growth and further support that Pin1 concentration is critical for HCC cell growth and migration.

### Genetic or chemical inhibition of Pin1 blocks multiple cancer-driving pathways simultaneously in HCC

**Figure 5:**
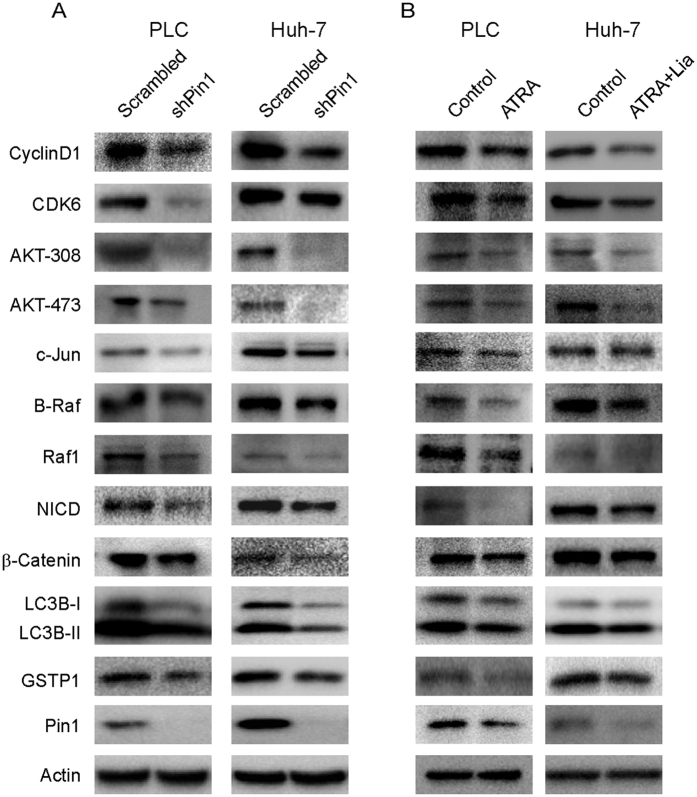
Both genetic and chemical Pin1 inhibition blocks multiple cancer-driving pathways simultaneously in human HCC cells. PLC/PRF/5 or Huh-7 cell lines were infected with lentivirus expressing scrambled or Pin1 shRNA (**A**), or treated with 10 μM ATRA (PLC/PRF/5) or 10 μM ATRA combined with 25 μM liarozole (Huh-7) (**B**). Cell lysates were subjected to Western blot analysis with specific antibodies.

Multiple cancer-driving pathways can be activated in the highly heterogeneous HCC^34^,^35^,^36^,^37^. As a major regulator of oncogenesis, Pin1 activates more than 40 tumor-promoting regulators and inhibits more than 20 tumor-suppressive regulators^12^. Since Pin1 might regulate different cancer substrates in different cancer types^12^, we asked whether inhibition of Pin1 by genetic or chemical ablation would affect protein levels of a selected set of Pin1 substrates or downstream factors, whose protein stability and abundance have been shown to be regulated by Pin1. We found that many growth promoting factors such as CyclinD1^47^, CDK6, pAKT^48^, c-Jun^49^, B-Raf, Notch intracellular domain NICD^50^, and β-catenin^51^ were significant decreased after Pin1 knockdown, both in Huh-7 and PLC/PRF/5 cells (Fig. 5A). Interestingly, LC3B, an autophage marker, and detoxification enzyme GSTP1 were also significantly changed (Fig. 5A). More importantly, a similar inhibition of multiple Pin1 downstream cancer-driving pathways was also found after chemical inhibition of Pin1 by its inhibitor ATRA (Fig. 5B). Thus, inhibition of Pin1 by chemical inhibitors or genetic knockdown has a unique property to inhibit multiple cancer-driving pathways at the same time in the complex cancer type HCC.

### Slow-releasing Pin1 inhibitor ATRA exerts potent anticancer activity against HCC *in vivo*

**Figure 6:**
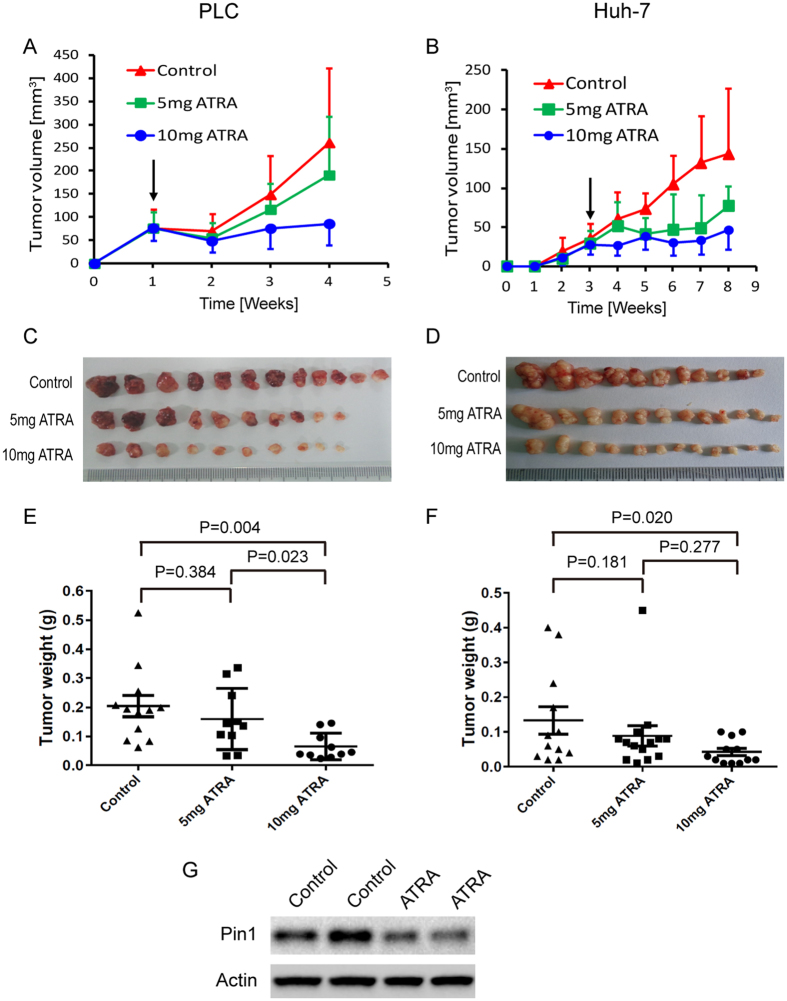
Slow-releasing Pin1 inhibitor ATRA potently inhibits HCC growth in mice. 2 × 10^5^ PLC/PRF/5 cells together with matrigel or 2 × 10^6^ Huh-7 cells were inoculated subcutaneously into nude mice. When tumors were obvious (tumor size 30–70 mm^3^), mice were randomly grouped to receive 5 or 10 mg 21-day slow-releasing ATRA or placebo pellets embedded under the neck skin. (**A**,**B**) PLC/PRF/5 (**A**) or Huh-7 (**B**) tumor volumes were measured weekly and the tumor growth curves were plotted over time. Error bars represent standard deviations. (**C**,**D**) Photographic illustration of PLC/PRF/5 (**C**) or Huh-7 (**D**) tumor nodules harvested from nude mice at the end point. Each scale of the ruler represents 1 mm. (**E**,**F**) Weights of Huh-7 (**E**) or PLC/PRF/5 (**F**) tumors harvested from nude mice at the end point. Error bars represent SEM. (**G**) Immunoblots of Pin1 expressed in xenograft tumors from nude mice inoculated with 4 × 10^6^ HuH7 cells and treated with placebo or 5 mg 21-day ATRA slow-releasing pellet for 3 weeks.

Given the obvious effects of ATRA on Pin1 protein levels, multiple cancer-driving pathways and cell growth in HCC *in vitro*, a critical question is whether ATRA suppresses HCC tumor growth *in vivo*. We thus examined ATRA in mouse models xenografted with HBV-positive PLC/PRF/5 and HBV-negative Huh-7 cells. ATRA is extremely light-sensitive and can be metabolized quickly in the liver, with a half life of 45 minutes in humans^33^,^52^,^53^. To improve drug activity against HCC, we implanted slow-releasing ATRA pellets, which maintain a constant drug level in the blood, as described previously^22^. 2 × 10^5^ PLC/PRF/5 together with matrigel or 2 × 10^6^ Huh-7 cells were injected subcutaneously onto both flanks of nude mice to generate tumors. When tumor volumes reached 60 mm^3^ in PLC/PRF/5 xenograft model or after 3 weeks in Huh-7 model, mice were randomly grouped to receive 5 or 10 mg 21-day slow-releasing ATRA pellets or placebo pills implanted under the skin in the back of the neck of each mouse, followed by measuring tumor growth every week. The tumor growth was potently suppressed by ATRA treatment in a dose dependent manner in PLC/PRF/5 model. As revealed by tumor growth curves or final tumor weights, while 5 mg slightly inhibited HCC tumor growth (P = 0.384), 10 mg ATRA completely blocked the tumor growth (P = 0.004) (Fig. 6A,C,E). Similar results were obtained in Huh-7 tumor model, with tumor inhibitory effects in 5 mg ATRA treatment group (P = 0.181) and more potent in 10 mg treatment group (P = 0.020) (Fig. 6B,D,F). The body weight of adult mice didn’t change during drug treatment, indicating a low cytotoxicity of ATRA at therapeutic dosage (data not shown). As expected, Pin1 protein was also downregulated in xenograft tumors from nude mice treated with slow-releasing ATRA (Fig. 6G). These results demonstrate for the first time that ATRA has potent anti-tumor activity against HCC through targeting Pin1.

## Discussion

We systematically evaluated the importance of Pin1 in HCC growth using genetic Pin1 knockdown and chemical Pin1 inhibition. Pin1 knockdown significantly inhibits both cells proliferation and tumorigenesis in various HCC cell lines *in vitro* and *in vivo*. Slow-releasing Pin1 inhibitor ATRA exhibits very potent inhibitory effects on HCC growth both *in vitro* and *in vivo*. Mechanistically, both genetic and chemical inhibition of Pin1 downregulates multiple cancer-driving pathways. Thus, Pin1 represents a novel promising therapeutic target for treating highly heterogeneous cancer HCC.

As the organ of metabolism and detoxifying, liver is exposed to many chemicals including carcinogens. HCC is a highly heterogeneous tumor in which cancer cells are able to use redundant pathways to escape from specific target/pathway blocking by drug treatment^34^,^35^,^36^,^37^. Although HCC is one of the most prevalent and malignant cancers, there are not many options for patients to choose against this disease^54^. As the only approved molecular therapeutic drug, Sorafenib only extends overall survival of patients by 2.8 months^3^. There is an urgent need to find novel efficacious therapeutics to control this disease.

As a global regulator of phosphor-proteins with Ser/Thr-Pro motif, Pin1 regulates multiple cancer-driving pathways in various HCC cell lines, making Pin1 as an ideal target for treating this heterogeneous cancer. Pin1 inhibitor ATRA is used in clinical to treat APL, but shows detectable but not striking anticancer activity against solid tumors^30^,^31^,^32^, very likely due to its light sensitivity and short half life of 45 min in humans^33^,^52^,^53^. By improving its stability in packaged slow-releasing pellet, we have shown for the first time a very potent anticancer activity of ATRA against HCC. As an acid form of vitamin A, ATRA only exhibits minor side effects on animals at experimental dose. In contrast, we noticed more severe side effects of Sorafenib in a side by side comparison experiment (unpublished data). Thus ATRA has unique advantages over current drugs, with characteristics of targeting multiple cancer-driving pathways and low side effects.

ATRA mimics the pSer/Thr-Pro motif in Pin1 substrates and its carboxylic and aromatic moieties can bind to the substrate phosphate- and proline-binding pockets of the Pin1 active site, respectively, thereby inhibit Pin1 catalytic activity and somehow induce Pin1 degradation^22^. Potentially due to high metabolism of ATRA in Huh-7 cells, ATRA alone could not induce Pin1 degradation efficiently *in vitro*. By blocking ATRA metabolism pathway, Cytochrome P450 inhibitor liarozole significantly enhances ATRA activity of inducing Pin1 degradation *in vitro*. This indicates that liarozole and ATRA combination might have additive effects on HCC therapy *in vivo*.

ATRA is light sensitive and unstable *in vivo*, which significantly decreases its activity against solid tumors. Although regular ATRA has moderate but detectable efficacy against solid tumors in some clinical trials, new generations of supposedly much more potent retinoid derivatives targeting their receptors RARs or RXRs show little efficacy^30^,^31^,^55^,^56^,^57^, which is likely due to the failure of these retinoids to inhibit Pin1^22^. Structural modification of ATRA molecule based on the Pin1-ATRA co-crystal structure^22^ with improved stability needs to be developed to increase ATRA anticancer activity.

We have shown a potent anticancer activity when using slow-releasing ATRA pellets to treat mice bearing HCC tumors. In contrast to free ATRA, these pellets are able to maintain ATRA serum concentrations in mice constant at the concentrations for Pin1 binding and inhibition as described previously^22^. We have packaged ATRA in slow-releasing microparticles, which alone shows more potent inhibitory activity both on Pin1 degradation *in vitro* and HCC tumor growth *in vivo* (data unpublished), indicating that developing novel slow-releasing method applicable to patients is worth further exploring. In addition, a combination of ATRA with current clinical drugs, such as sorafenib, paclitaxel, 5-Fu might have additive/synergistic therapeutic effects and represents a promising strategy for treating HCC.

In summary, our results have shown for the fist time that targeting Pin1 offers a promising therapeutic approach to simultaneously stop multiple cancer-driving pathways in HCC, further providing a rationale for developing longer half-life ATRA or more potent and specific Pin1-targeted ATRA derivatives to overcome drug resistance in treating aggressive cancers such as HCC.

## Materials and Methods

### Cell lines, antibodies and animals

Cell lines Huh-7 and PLC/PRF/5 were bought from Cell Bank of Chinese Academy of Sciences. Cells were maintained in DMEM media (#12800017; GIBCO) supplemented with 10% fetal bovine serum (#10437-028; GIBCO) and 1.5 g/L of NaHCO_3_. The protein samples were blotted with following antibodies: rabbit anti-Cyclin D1 (#2978 S; Cell Signaling Technology) 1:1000, mouse anti-CDK6 (#3136 S; Cell Signaling Technology) 1:1000, rabbit anti-Phospho-Akt(Thr308) (#9275 S; Cell Signaling Technology) 1:600, rabbit anti-Phospho-Akt(Ser473) (#9271 S; Cell Signaling Technology) 1:600, rabbit anti-c-Jun (60A8) (#9165 S; Cell Signaling Technology) 1:1000, rabbit anti-B-Raf (#sc-166; Santa Cruz Biotechnology) 1;400, rabbit anti-Cleaved Notch1 (Val1744) (#4147 S; Cell Signaling technology)1:300, rabbit anti-β-Catenin (#8480 S; Cell Signaling technology) 1:1000, rabbit anti-LC3B (#ab48394; abcam) 1:1000, mouse anti-GSTP1 (#3369 S; Cell Signaling technology) 1:1000, mouse anti-Actin (#HC201; TransGen Biotech) 1:3000. BALB/c nude mice were housed in laminar flow cabinets with free access to food and water in Laboratory Animal Center of Fujian Medical University. All of animal experiments were performed in accordance with the animal protocols and regulations approved by FJMU Experimental Animal Ethics Committee of Fujian Medical University.

### Tumor implantation and drug treatment

5 × 10^5^ PLC/PRF/5 cells together with matrigel (#356231; BD) or 2 × 10^6^ Huh-7 cells were inoculated subcutaneously onto the left and right flank region of ~5-week old nude mice. Tumor volume was measured every week and calculated by the formula: length × width^2^/2. For Pin1 knockdown experiments, cells were infected by lentivirus expressing scrambled or Pin1 shRNA, selected by drug puromycin, expanded and inoculated onto nude mice at early passage (<6 passages).

For ATRA treatment, ATRA slow-releasing pellet (#V-111; Innovative Research of America) was embedded under the neck skin of mice 3 weeks after tumor cells inoculation (Huh-7) or when tumor volume reaches 60 mm^3^ (PLC/PRF/5) using Gauge Precision Trochar. It is normal that minor inflammation occurs around the drug pellet. It should be noted that anesthetic drug chloral hydrate together with ATRA pellet evokes very severe inflammation, which would complicate the results. Instead, anesthetic drug ketamine has no this problem.

### Transwell assay

Hanging cell culture insert with pore size of 8.0 μm (#3442, Corning) were coated with 0.6 μg of collagen (#C4243; Sigma-Aldrich) and placed onto 24-well plate. 200 μl of 5 × 10^4^ HCC cells starved O/N in media with 1% FBS were placed in the upper chamber and 500 ml of media with 10% PBS were placed in the lower chamber. The plate was placed in incubator to allow cell migration for 36 hrs. The remained cells in the upper chamber were removed using cotton swab and the inserts were fixed by methanol followed by staining with 0.1% crystal violet for 20 min. Migrated cells adhering to the underside of the inserts were photographed for 10–20 fields and counted under a light microscope at x200 magnification. For drug treatment experiments, cells were pretreated with ATRA, liarozole or combination of ATRA and liarozole for 24 hrs and were incubated with drugs during the whole transwell process.

## Additional Information

**How to cite this article:** Liao, X.-H. *et al*. Chemical or genetic Pin1 inhibition exerts potent anticancer activity against hepatocellular carcinoma by blocking multiple cancer-driving pathways. *Sci. Rep.* **7**, 43639; doi: 10.1038/srep43639 (2017).

**Publisher's note:** Springer Nature remains neutral with regard to jurisdictional claims in published maps and institutional affiliations.
